# Dynamic change of glomerular filtration rate in the early stage is associated with kidney allograft status: a preliminary report

**DOI:** 10.1186/s40001-014-0072-6

**Published:** 2014-12-24

**Authors:** Guisheng Qi, Qunye Tang, Ruiming Rong

**Affiliations:** Department of Urology, Zhongshan Hospital, Fudan University, 180 Fenglin Road, Shanghai, 200032 People’s Republic of China; Shanghai Key Laboratory of Organ Transplantation, Shanghai, People’s Republic of China; Department of Transfusion, Zhongshan Hospital, Fudan University, Shanghai, People’s Republic of China

**Keywords:** Estimated glomerular filtration rate, Slope, Interstitial fibrosis and tubular atrophy, Renal transplantation

## Abstract

**Background:**

This study aimed to investigate the relationship between the dynamic changes of estimated glomerular filtration rate (eGFR) in the early stage post renal transplantation and renal allograft dysfunction.

**Methods:**

We selected 9 patients with interstitial fibrosis and tubular atrophy (IF/TA) and 11 patients with stable renal function based on the Banff 2007 classification system. Pathology of the patients was evidenced with renal biopsy results. Glomerular filtration rate (GFR) was calculated continuously for 14 days post-transplantation by using an estimated GFR (eGFR) formula adjusted into Chinese. Linear regression was employed, and eGFR slopes were compared. Prisoners or organs from prisoners were not used in this study.

**Results and Conclusion:**

The eGFR slope in the IF/TA group was significantly higher than that in the stable group (*P* < 0.01*),* and a cut-off value of 5.11 mL/min/1.73 m^2^/d was a reliable clinical value in a receiver operating characteristic (ROC) curve. On the basis of the ROC area under the curve, predictive accuracy of the eGFR slope was excellent (0.848). In conclusion, the eGFR in IF/TA increased faster within a period of 14 days post-transplantation, suggesting that reperfusion in the early stage may damage the glomerular filtration membrane to some extent. Furthermore, reperfusion might adversely affect long-term renal allograft survival.

## Background

Kidney transplantation is the standard treatment for patients with end-stage kidney disease. Acute rejection rates and early graft loss have decreased substantially over the past four decades. However, progressive chronic allograft dysfunction, particularly interstitial fibrosis and tubular atrophy (IF/TA), remains a common cause of late graft loss [1-5]. Early prognosis of a kidney transplant is critical for the management of subsequent therapy. A considerable number of risk factors have been identified to influence short- and long-term graft survival, such as recipient and donor age, presence of diabetes mellitus, human leukocyte antigen mismatch, prolonged cold ischemia time, cytomegalovirus infection, acute rejection episodes, and delayed graft function [6-8]. In the early post-transplantation stages, several important clinical factors may influence kidney graft function, such as blood residues, rejection episodes, and acute immunosuppressive drug toxicity.

For decades, serum creatinine (Scr) has been a critical clinical parameter that is widely used to evaluate the function of transplanted kidneys. However, Scr values are abnormal only in severe renal dysfunction. Thus, Scr cannot be used to detect early stages of renal disorders. The most reliable method for evaluation of renal function is measurement of GFR [9,10]. In this study, we evaluated the relationship between prognosis and eGFR changes in the early stage post renal transplantation. Moreover, the eGFR slope was evaluated for sensitivity and specificity by using the receiver operation characteristic (ROC) curve.

## Methods

### Baseline characteristics of the patients

From January to December 2012, 20 living related renal transplanted recipients, whose protocol biopsy results were either normal (stable) or IF/TA, were enrolled in this retrospective study. Biopsy was performed 1 year post-transplantation, and results were defined by the Banff 2007 classification [11]. Other key inclusion criteria were panel reactive antibodies (PRA) < 20% on the day of transplant and/or before transplant, first-time renal transplantation, and over 1 year post-transplantation. Patients who received a multi-organ transplant, had undergone long-term immunosuppression before transplantation, were experiencing generalized infection at the time of transplant, had a history of malignancy, or were positive for HIV, HCV antibody, or HbsAg were excluded from the study. All these living related renal transplantat patients and this study were approved by the Ethics Committee of Zhongshan Hospital, Fudan University (Shanghai, China). Procedures in this study were in accordance with the Helsinki Declaration of 1975. No prisoners or organs from prisoners were used in this study. Informed consents were obtained from these patients and living related donors.

### eGFR formula

The eGFR formula was based on the Modification of Diet in Renal Diseases (MDRD) formula and was adjusted to Chinese and called c-aGFR:

$$ \mathrm{c}-\mathrm{aGFR}\ \left(\mathrm{mL}/ \min /1.73\ {\mathrm{m}}^2\right) = \left[186 \times \mathrm{S}\mathrm{c}\mathrm{r}\right] - 1.154 \times \left[\mathrm{Age}\right] - 0.203 \times 0.742\ \left(\mathrm{Female}\right) \times 1.233 $$

[12] c-aGFR was calculated for 14 days post-transplantation.

### Statistics

Results were expressed as mean values ± SD. IBM SPSS 19.0 (International Business Machines Corp., Armonk, NY, USA) was used for data analysis. At baseline, proportion gender ratio, primary diagnosis, and immunosuppressive protocol were compared by Chi-square test of independence. Mean age, body mass index, cold/warm ischemia time, and pre-transplant PRA levels were compared by two-tailed Student's *t*-tests. All other variables were presented descriptively. The slope of eGFR was calculated by linear regression. Two sample unpaired *t*-tests were used to compare eGFR slopes between stable and IF/TA patients. The area under the curve (AUC) of ROC was calculated to evaluate sensitivity and specificity. Overall, *P* < 0.05 was regarded as significant.

## Results

### Characteristics of the transplant recipients

Table 1 shows the characteristics of patients. Table 2 lists the grade of IF/TA according to Banff 2007 for each patient in the IF/TA group.

**Table 1:** **Demographic characteristics of patients**

	**Stable**	**IF/TA**	***P*** **-value**
Numbers	9	11	
Gender (male/female)	4/5	5/6	>0.05
Age (years)	42.75 ± 3.51	43.22 ± 2.94	>0.05
Weight (kg)	53.11 ± 3.24	54.09 ± 4.76	>0.05
BMI	23.98 ± 1.98	24.08 ± 1.72	>0.05
Cold ischemia time (min)	36.98 ± 0.81	37.02 ± 0.91	>0.05
Warm ischemia time (min)	4.91 ± 0.55	5.01 ± 0.40	>0.05
Primary diagnosis			
Glomerulonephritis	7	8	>0.05
Nephrotic syndrome	2	3	>0.05
Pre-transplant PRA (%)			
Class I	1.08 ± 0.87	0	>0.05
Class II	1.11 ± 0.80	0	>0.05
ISP			
CsA + MMF + Pred	6	7	>0.05
Tac + MMF + Pred	3	4	>0.05

IF/TA: interstitial fibrosis and tubular atrophy; BMI: body mass index; PRA: panel reactive antibody; ISP: immunosuppressive protocol; CsA: cyclosporine A; Tac: tacrolimus; MMF: mycophenolate mofetil; Pred: prednisone. Data were presented as mean values with standard error of the mean.

**Table 2:** **Interstitial fibrosis and tubular atrophy (IF/TA) grade for each patient in the IF/TA group**

**Patient number**	**Grade**
1	I
2	I
3	III
4	II
5	III
6	II
7	I
8	II
9	II
10	I
11	III

### Comparison of eGFR slope

**Figure 1:**
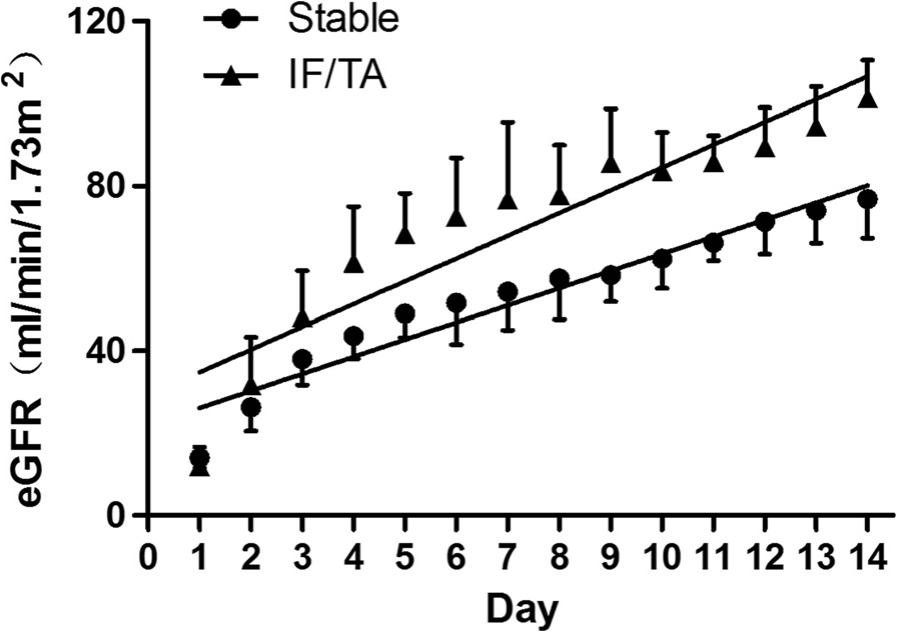
**Linear trend of calculated estimated glomerular filtration rates (eGFRs) in each post-transplant day.** The slope in the interstitial fibrosis and tubular atrophy (IF/TA) group was significantly higher than that in the stable group.

The linear trend of calculated eGFRs in each day post-transplantation was analyzed in both stable and IF/TA groups. The slope in the IF/TA group is significantly higher than that in the stable group (5.52 ± 0.29 versus 4.15 ± 0.19) (Figure 1).

### Renal function

**Figure 2:**
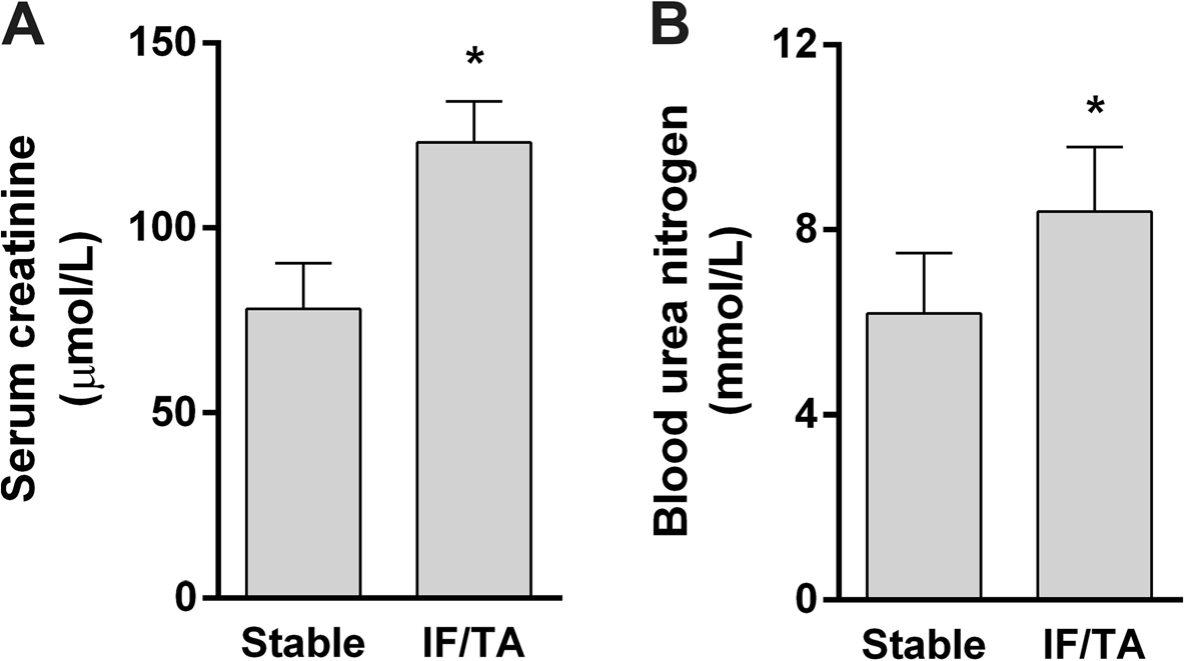
**Renal function when the biopsy was performed.** Both the serum creatinine (Scr) **(A)** and blood urine nitrogen (BUN) **(B)** in the interstitial fibrosis and tubular atrophy (IF/TA) group were significantly higher than those in the stable group.

The Scr and blood urine nitrogen (BUN) were tested when biopsy was performed. Both the Scr and BUN in the IF/TA group were significantly higher than those in the stable group (Figure 2).

### The ROC curve

**Figure 3:**
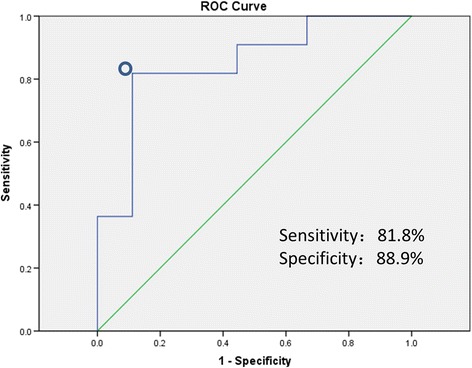
**Receiver operating characteristic curve.** The circle indicates the cut-off value of 5.11 ml/min/1.73 m^2^/d with 81.8% sensitivity and 88.9% specificity, and the area under the curve is 0.848.

The ROC curve was determined to differentiate IF/TA patients from stable patients. The eGFR slope value of 5.11 mL/min/1.73 m^2^/d was the cut-off value of 0.848 (95% CI, 0.672 to 1.000, *P* < 0.01, Figure 3). The cut-off value exhibited a sensitivity of 81.8% and specificity of 88.9% in the identification of IF/TA patients, suggesting a potential clinical value and excellent ROC AUC (0.848).

## Discussion

This study is the first to analyze eGFRs obtained on a daily basis for 14 days post-transplantation using linear regression. We found that the eGFR slope in IF/TA patients was significantly higher than that in stable patients. The eGFR slope value of 5.11 mL/min/1.73 m^2^/d as a cut-off value provided a sensitivity of 81.8% and specificity of 88.9% in the identification of IF/TA patients. Results revealed that the slope in the early stage post-transplantation may be an indicator of 1-year allograft dysfunction.

The eGFR is a better alternative parameter than creatinine in the evaluation of renal function in post-transplanted kidneys. Increasing eGFR in the early stage post-transplantation reflects recovery of renal function. However, the rate may not be favorable. The result showed that the rapid increase in eGFR resulted in adverse effects after 1 year. This interesting result may be attributed to early reperfusion overload in transplanted kidneys. Previous studies demonstrated that mechanical stress in the renal artery could lead to severe injury of the renal graft, independent from immune factors such as rejection or inflammation. Tovbin *et al*. reported a case wherein rapid reperfusion occurred because of left axillo-femoral bypass graft surgery and induced progressive glomerulonephritis in a renal transplant patient [13]. Aggravated capillary damage, inflammation, and oxidative stress following successful reperfusion are possible explanations for the case. Putative mechanisms for these phenomena are interaction of reperfusion-induced hyperfiltration, high intraglomerular capillary pressure, oxidative stress, increased polymorphonuclear cell infiltration, and inflammation [13,14]. In the early stage post renal transplantation, the glomerular filtration membrane is constantly exposed to high pressure for several days, which may lead to proteinuria and potential tissue injury. Given that pathological examination is the standard method to evaluate the long-term outcome of a renal graft, patients in this study were divided into stable and IF/TA groups according to the protocol biopsy results. We hypothesize that immediate overload in the reperfused kidney is a critical direct risk factor in chronic graft deterioration.

eGFR is a reliable reflection of perfusion loading and recovery of the transplanted kidney. In this study, the eGFR was calculated according to the Chinese-adjusted abbreviated MDRD formula (c-aMDRD) for a more accurate GFR estimation for Chinese people compared with Nankivell or Cockcroft-Gault formula, or typical aMDRD formula [12,15,16]. In the long run, increasing eGFR in the post-transplant period indicates recovery of renal graft function. However, the extremely rapid rise of eGFR may not necessarily be favorable. We analyzed the eGFR of patients within 14 days after renal transplantation and examined the linear relationship of day-by-day eGFRs in both groups. Results exhibited a positive linear fit among the eGFR values with time. Slopes obtained through calculations may reflect the rate of eGFR increase and thus indicate graft perfusion load. The slope in the IF/TA group was significantly higher than that in the stable group, which was in accordance with our expectation.

We finally formulated hypotheses that may be involved in over-perfusion within the renal graft in the early stage after transplantation:

1. Sequence of opening of renal artery and vein at reperfusion. As one of the high perfusion organs, the kidney can filter nearly one third of the circulation volume within 1 minute. If the renal artery is opened first, the inner pressure of the kidney will rapidly reach the peak, which may injure the glomerular filtration membrane. Ideally, the renal artery and vein should be opened simultaneously. However, this procedure is difficult in practice. In our opinion, opening the renal vein immediately before opening the renal artery is safer to ensure the stability of intra-renal pressure, which may protect the glomerular filtration membrane.
2. Ligation of the minor branches of the vein. The conservation of arterial branches is a well-accepted procedure for enabling sufficient perfusion of the kidney. However, the conservation of small venous branches remains controversial. In some instances, ligated branches collect a large reservoir of blood in the kidney, which may result in high intra-renal pressure that injures the glomerular filtration membrane, even when the general blood pressure is normal.
3. Blood pressure level during perioperative period. In general, patients with end-stage chronic renal failure suffer from hypertension. This condition requires specific attention during the operation, especially before opening the vasculature. Blood pressure may vary during the perioperative period. Blood pressure for optimal recovery of kidney function is difficult to establish. Some studies report an optimal pressure ranging from 80 mmHg to 125 mmHg, whereas others claim that the systolic pressure should not be lower than 140 mmHg to avoid insufficient perfusion of the transplanted kidney [17,18]. In uremia patients, long-term hypertension, anemia, or atherosclerosis resulted in vascular smooth muscle dysfunction, which may further deteriorate the self-regulation of renal vessels. Therefore, we recommend the use of vasoactive agents when necessary in the early post-operative stage for the maintenance of appropriate renal perfusion pressure. When the patient has suffered from hypertension for many years, the administration of an anti-hypertensive agent is highly recommended to protect the renal graft as early as possible [19,20].

The limitations in this study should be noted. First, the sample size was small. Second, it would be very helpful to compare the surgical technique data such as the opening sequence of artery and vein, the number of ligated free veins and blood pressure fluctuation in perioperative period between the two groups. We are now, however, unfortunately unable to access the original data. Moreover, long-term follow-up is required to find a cut-off eGFR value as a prognostic reference.

## Conclusion

In conclusion, a relationship exists between early eGFR variation after renal transplantation and prognosis. The rapid elevation of eGFR reflects high reperfusion, which may have disadvantages for prognosis. Therefore, in clinical settings, the blood pressure should be maintained within a certain range during or immediately after transplantation to decrease the damage caused by high reperfusion to the glomerular filtration membrane and to ameliorate the prognosis.

## Abbreviations

eGFR: estimated glomerular filtration rate
ROC: receiver operating characteristic
IF/TA: interstitial fibrosis and tubular atrophy
Scr: serum creatinine
PRA: panel reactive antibodies
MDRD: Modification of Diet in Renal Diseases
BUN: blood urine nitrogen
